# Persistent and transient productive efficiency in the African airline industry

**DOI:** 10.1007/s11123-023-00674-3

**Published:** 2023-05-26

**Authors:** Gianmaria Martini, Flavio Porta, Davide Scotti

**Affiliations:** Università degli studi di Bergamo, Bergamo, Italy

**Keywords:** Persistent and transient efficiency, African airlines, Stochastic frontier analysis

## Abstract

Airline efficiency growth is considered one of the key factors for aviation sustainability in Africa and for creating a successful relationship between aviation activities and economic development in the continent. This paper proposes estimating the efficiency of African airlines in the period 2010–2019 using a state-of-the-art stochastic frontier model disentangling persistent efficiency, transient efficiency, and unobserved heterogeneity. We also examine the impact on both persistent and transient efficiency of (i) ownership structure, (ii) political stability, (iii) airline geographical location, (iv) airline domicile country’s economic freedom, and (v) airline participation in a global alliance. We find evidence of relatively low efficiency and decreasing returns to scale, implying that it is important to achieve better utilization of inputs. Our findings also suggest that protectionism seems still an important driver of efficiency in a context characterized by a lack of liberalization. However, enhanced economic freedom is found to be more relevant in improving the efficiencies of African airlines, suggesting that policy interventions aimed at speeding up the liberalization process may help to remove the conditions that make air carriers operate inefficiently.

## Introduction

It is generally agreed in the literature that the supply-side transportation infrastructure fosters economic development and that this effect is greater the more solid and efficient the airline industry connected to it. Many studies provide evidence of the relationship between air transport services and regional development. For example, air transport services proved to positively influence (i) the growth of population and employment levels (Blonigen and Cristea 2012, Green 2007), (ii) tourist activities (Graham and Dobruszkes 2019), (iii) agglomeration economies (Glaeser et al. 1992, Rosenthal and Strange 2001), (iv) foreign direct investment flows (Fageda 2017), and (v) international trade (Button et al. 2015).

This connection is crucial for Africa, which is the largest continent on earth, with many landlocked countries, and poor road and railways infrastructures. Unfortunately, African airlines, especially in Sub-Saharan Africa, notoriously suffer from a lack of efficiency due to several reasons. They are relatively small, enjoy little economies of density and scope, face market instability and lack of liberalization, are often subject to considerable political interference, and are characterized by a lack of cooperation (Button et al. 2017, Button 2022). This explains why airline efficiency growth is considered one of the main paths ahead for aviation sustainability in Africa and, in turn, for creating a successful relationship between aviation activities and economic development in the continent (ADBG 2019).

A key factor toward economic development, especially in such a context of an underdeveloped aviation industry, is represented by policy interventions aimed at removing the conditions that make airlines operate inefficiently. In this regard, Africa is lagging behind other regions in the world like the US and Europe since the deregulation process is still far from being completed, as discussed in detail in Section 3.

This paper proposes estimating the efficiency of African airlines in the period 2010-2019 using a recently developed parametric method (Colombi et al. 2014, 2017) and examining the impact of a set of possible determinants on the estimated scores. More knowledge about the efficiency of the continent’s carriers and its determinants is expected to provide African governments and policymakers useful information in improving the industry and consequently enjoying the associated wider economic benefits, especially in the light of the COVID-19 pandemic, whose impact on African airlines has been severe (UNECA 2020).

The paper is organized as follows: Section 2 revises previous contributions on airlines efficiency, Section 3 presents the main features of the African airline industry. Section 4 presents the empirical model, while Section 5 describes the data and provides some descriptive statistics. Section 6 shows our results, Section 7 performs the diagnostic checks on the microeconomic foundations of the estimated production function, while Section 8 provides additional evidence coming from alternative model specifications. Section 9 concludes the paper with some policy implications, while we report an additional figure in the Appendix at the end of the paper.

## Literature review

Since the 1980s the transportation economics literature studies airline performances with a focus mainly on technical efficiency and total factor productivity (Scotti and Volta 2017). Heshmati and Kim (2016) and Yu (2016) provide a detailed review of the methodologies and the variables used in this kind of studies. Traditionally, researchers are mainly focused on the factors affecting efficiency and on how technical efficiency and productivity evolve over time (Alam and Sickles 1998, Good et al. 1993, 1995, Oum and Yu 1995, Sickles et al. 2002). Some other benchmarking studies investigate airline cost efficiency (Heshmati et al. 2018, Oum and Yu 1998, Oum and Zhang 1991), productivity and cost competitiveness (Oum and Yu 2012, Windle 1991), or airline profitability (Scotti and Volta 2017).

Looking specifically at studies focused on technical efficiency (i.e., the subject of our paper), they apply both Data Envelopment Analysis (DEA) and Stochastic Frontier Analysis (SFA). The properties of the two approaches are well known by researchers as well as their advantages and disadvantages. Coelli et al. (2005) explains in detail that DEA, as a non-parametric and deterministic approach, does not require any assumption on the functional form of the production function, but measurement errors and other sources of statistical noise are basically ignored. On the contrary, SFA estimates the frontier parametrically thanks to the introduction of a random component error term that captures statistical noise. This requires assumptions (i) on the functional form of the production function under study, and (ii) on the statistical distribution of the error term. From the methodological point of view, our paper belongs to the group of parametric studies and applies a quite recent SF model (Colombi et al. 2014, 2017) that, as explained in detail in the methodology section, has two main advantages: (i) it avoids confounding time-invariant inefficiency with unobserved heterogeneity, and (ii) it allows to disentangle persistent (long-run/time-invariant) from temporary inefficiency (short-run/time-varying). To the best of our knowledge, the only contribution distinguishing between persistent and transient efficiency applied to the airline industry is Heshmati et al. (2018). However, this paper is focused on international airlines and cost efficiency, and it applies an estimation method based on Filippini and Greene (2016) approach, which is a simulated maximum likelihood estimation method. No African airlines are included in the data set.^1^Filippini and Greene (2016) exploits the possibility to characterize the four random component model as a pair of two-part disturbances in which each element of the pair has its own skew-normal distribution, and this is a less general approach than the method developed by Colombi et al. (2014, 2017) and adopted here. In a trade-off between statistical efficiency and estimation time Filippini and Greene (2016) might be useful when the ML estimation method becomes computationally demanding, i.e., for a long time horizon.^2^

Concerning the variables used in the literature on airline efficiency, and looking at inputs, it is quite common to observe studies focusing on labor and capital, sometimes combined with materials or energy. It is not uncommon also to find, among the inputs selected, monetary variables such as operating costs or fuel expenses. In terms of outputs, the most used variables are passengers, freight, and revenue passenger kilometers, but also monetary variables such as revenues. Among the factors affecting efficiency, the most considered variables are (i) ownership structure, (ii) fleet characteristics, (iii) network characteristics, and (iv) business-model-related variables such as alliance membership and being a low-cost carrier.

If we look more specifically at the papers on African airlines, apart from some contributions that apply benchmarking analysis to a set of data that includes some of the world’s major airlines and takes also into account African carriers (e.g., Aydın et al. 2020, Merkert and Hensher 2011), our work is more connected to papers studying efficiency using data only for African carriers. Hence, there are only two previous contributions: Barros and Wanke (2015) use the Technique for Order Preference by Similarity to the Ideal Solution (TOPSIS), namely a multiple-criteria decision-making method, to rank 29 African carriers for the period 2010–2013. The inputs considered are the number of employees, the number of aircraft (as a proxy for capital), and the operating costs. The two output variables used are revenue passenger kilometers and revenue tonne-kilometers. The authors also perform a second-stage analysis based on neural network techniques, where they consider as contextual variables some business characteristics (e.g., the age of the company and the public ownership), network size (number of domestic, intra-African, and international destinations), and the fleet mix in terms of aircraft models. They find on average a low-efficiency level in African airlines. They also find a positive impact of public ownership on efficiency scores suggesting a linkage between performance and protectionist practices adopted by African governments. Barros and Wanke (2016) adopt instead a two-stage network DEA approach to analyze the same data used in Barros and Wanke (2015). The novelty there is that the production process of African carriers is decomposed into a first stage, where employees and aircraft are the input variables used to produce destinations (the efficiency of this sub-process is called “network efficiency”), and a second stage where destinations are used to produce financial revenues. The resultant efficiency scores exhibit little variation over time at the airline level, and the average efficiency scores are confirmed low, with revenue efficiency lower than network efficiency. Concerning environmental factors, public ownership plays a negative role in network efficiency, but a positive role in revenue generation. Years in business are found to affect positively efficiency, while the relevance of airline fleet mix varies depending on the specific aircraft model considered.

Finally, some papers focus on South Africa only, namely Mhlanga (2019, 2020), Mhlanga et al. (2018). Despite the reduced sample, in terms of both geographical focus and size (few airlines and a limited number of years), these papers highlight once again the relevance of the ownership structure as a driver of efficiency. More specifically, Mhlanga et al. (2018) and Mhlanga (2019) benchmark ten airlines in Southern Africa (period 2012–2016). They combine DEA to a second stage analysis based on a two-way random effects GLS and also Tobit regression, and show that public ownership negatively affects technical efficiency as a result of government veto power over the airlines’ commercial choices. Also, LCCs are found more efficient as well as airlines with bigger aircraft and higher load factors. Mhlanga (2020) analyze a sample of nine South African airlines in the period 2015–2018 with a bootstrapped meta-frontier approach. The paper confirms that airline ownership (together with aircraft size and airline cost structure) significantly affects technical efficiency. More specifically, public ownership is found to be negative for efficiency, in line with the previous contribution on South African airlines.

Taking into account that the African airline industry is under-investigated in comparison to more advanced industries like the US and the European ones, and this is especially true for efficiency studies, the main contributions of our paper are as follows: (i) to analyze African airline efficiency and some of its determinants through a quite advanced methodological parametric approach, never applied before to the African airline industry, and (ii) to extend the period of analysis to a longer time interval. Hence, our empirical findings may have interesting managerial and policy implications contributing to the sustainability of African aviation and reinforcing the relationship between airline services and economic development in African countries.

## The African airline industry

It is generally recognized that Africa has great potential for the development of air services. The continent represents a significant portion (about 15%) of the world’s population, spread in more than 50 countries, and its geography is characterized by huge distances and increasingly by large urban concentrations (Button et al. 2015, Lubbe and Shornikova 2017). Despite that, African continental airline markets are quite small (only about 2% of global traffic) and concentrated in a few countries, with most of the airlines that are locally oriented (Button et al. 2022). More specifically, African airlines are small, especially in Sub-Saharan Africa, and, on top of that, are often subject to considerable political interference. As a result, market instability is an issue in an industry that regularly observes airlines entering and exiting the market. Ssamula and Venter (2013) point out that the enormous size of the continent, its relatively low overall population density, the artificiality of many national borders, and the rapid spread of urbanization make the development of efficient transportation networks a challenge; specifically, it is difficult to develop Hub and Spoke (H&S) networks that are essential for airline profitability. A H&S network contributes to economies of density by concentrating passengers with different origins in a hub airport, to reach the same destination with a connecting flight. This generates larger volumes for the airlines (Bilotkach 2017).

The COVID-19 pandemic has made things even worse, with Africa constantly lagging behind other regions in terms of vaccine rates, and, in turn, large reductions in aviation activities (Andreana et al. 2021). As highlighted by IATA (2021), the pandemic (i) affected tourism and business travel volumes bringing an increase in airlines’ losses from −2.7 $/passenger in 2019 to −44.6 $/passenger in 2020, and slowing down the recovery (current IATA predictions for 2022 indicate a −21.8 $/passenger).

Apart from the COVID-19 pandemic, there are many reasons that may explain the bad economic performance of African airlines. First, the lack of liberalization. Globally, the liberalization of air passenger services began in the US (Airline Deregulation Act., 1978), followed by Europe (about a decade later). With a little delay, also in Africa, some efforts were made over the last 25 years toward the creation of a multilateral air transportation common market: the Yamoussoukro Decision (YD) in 1999 was the most significant agreement in this direction (Scotti et al. 2017). YD aimed at liberalizing international air travel within Africa, but it did not prove very successful (Lubbe and Shornikova 2017, Njoya 2016), mainly for political reasons. As a result, new efforts were required, and, in 2018, they materialized in the foundation of a Single African Air Transport Market (SAATM) aimed at accelerating the full implementation of YD (Button et al. 2022). SAATM is currently under implementation, even if it is still hindered by factors such as the culture of non-prioritization of aviation, and protectionist policies (InterVISTAS 2021). Other traditional reasons behind the poor performances of African airlines are high costs (compared to the rest of the world) of both fuel and airport charges, old aircraft fleets, the lack of a skilled labor force, and the competition from extra-African carriers (European and Gulf airlines). Button et al. (2022) identify also in the limited participation to a strategic global alliance by African airlines a potentially important reason of the latter’s underdevelopment. Joining an alliance can indeed bring advantages to airlines with regard, for example, to network coordination, scale economies, and, in the long run, access to new markets. Despite this, airline participation in global alliances is, in fact, limited to five carriers, i.e., *Egyptair*, *Ethiopian Airlines*, and *South African Airlines* (*Star Alliance*), *Kenya Airways* (*Skyteam*), and *Royal Air Maroc* (*oneworld*, but only as of 2020).

The difficulties of the aviation sector are a factor explaining the gap in economic growth between African countries and more developed economies; the existing literature (Antunes et al. 2020, Manello et al. 2022) indeed agrees on the positive impact exerted on economic growth by aviation development, with benefits observable in terms of trade volumes, income, employment, firm localization, and industrial relations.

The issue of airline efficiency is therefore extremely important in the African context, and this is even more important in the current context of recovery from the COVID-19 crisis. African governments appear more and more aware of the wider economic benefits associated with an efficient aviation industry and have now more than ever an important opportunity to rethink the future of their inefficient/unprofitable carriers. Indeed, only the most efficient and profitable airlines have a chance to withstand the current tide of passenger restrictions (Thomas 2020). Hence, it is crucial to identify efficient airlines and to understand the drivers of efficiency.

## Empirical model for African airline efficiency

Our aim is to estimate a production function for African airlines using an SFA model with transient efficiency, persistent efficiencies, and unobserved heterogeneity, as in Colombi et al. (2014), and in Colombi et al. (2017). We present now our baseline model, while alternative specifications are discussed in subsection 4.1. We consider the following airline production frontier model:

1 $${y}_{it}={\beta }_{0}+{{{{\boldsymbol{x}}}}}_{it}^{{\prime} }{{{\boldsymbol{\beta }}}}+{{{{\boldsymbol{f}}}}}_{it}^{{\prime} }{{{\boldsymbol{\theta }}}}+{b}_{i}-{u}_{it}-{u}_{i}+{e}_{it},$$

where the index *i* (*i* = 1, 2, …, *N*), denotes the *N* African airlines in the sample, and *t* (*t* = 1, 2, …, *T*, ) the *T* periods at which each airline is observed. The dependent variable *y*_*it*_ in our baseline framework is the logarithm of airline *i*’s annual number of passengers in period *t* (*PAX*); ${{{{\boldsymbol{x}}}}}_{it}^{{\prime} }$ is a row vector of *p* inputs involved in airline *i*’s production process (in our base model labor *L* and capital *K*) and ***β*** is a column vector of *p* unknown parameters. ${{{{\boldsymbol{f}}}}}_{it}^{{\prime} }$ is a vector of production shifters: as mentioned before, since the implementation of a H&S network may increase volumes, we will consider as a production shifter the centralization of airline *i*’s route network in period *t*. The adopted index of centralization is betweenness centralization (*BETWEEN*). The latter might capture how close an airline network is to a H&S structure; hence it may have an impact on traffic. Indeed, a H&S system serves more destinations than any alternative network system, being equal to the number of routes operated, and has implications in terms of market size (Button 2002, Cook and Goodwin 2008). For these reasons, we believe it has to be incorporated into the analysis as a potential shifter of the output level. How to compute betweenness centralization will be explained in Section 5. ***θ*** is a column vector of coefficients related to production shifter: since we only consider *BETWEEN*, in our empirical investigation is a scalar.

The random-airline effect *b*_*i*_ captures unobserved heterogeneity, *u*_*it*_ is a non-negative random variable for transient inefficiency of airline *i* at period *t*, *u*_*i*_ is a non-negative random variable for persistent inefficiency of airline *i*, and *e*_*it*_ is a normal random variable representing the exogenous shock affecting airline *i*’s output in period *t*. We assume that:

1. for *i* = 1, 2, …, *N*, and *t* = 1, 2, …, *T* the 2(*T* + 1) random variables *u*_*i*_, *b*_*i*_, *u*_*it*_, and *e*_*it*_ are independent in probability. This means that, for each airline, the random components in the model (1) are independent.
2. The random vectors (*b*_*i*_, *u*_*i*_, *u*_*i*1_, *u*_*i*2_, …, *u*_*iT*_, *e*_*i*1_, *e*_*i*2_, …, *e*_*iT*_), *i* = 1, 2, …, *N*, are independent in probability, i.e., the random components are independent among airlines.
3. For each *i*, *u*_*i*_ is a normal random variable, with mean *μ* and variance ${\sigma }_{ui}^{2}$, left-truncated at zero, and *b*_*i*_ is a normal random variable with null expected value and variance ${\sigma }_{b}^{2}$.
4. For each *i* and *t*, *u*_*it*_ is a normal random variable, with mean *λ* and variance ${\sigma }_{uit}^{2}$, left-truncated at zero, and *e*_*it*_ is a normal random variable that has null expected value and variance ${\sigma }_{e}^{2}$.
5. ${{{\boldsymbol{{x}}}_{it}^{{\prime} }}}$ are vectors of exogenous variables.

Hence, we assume that ${e}_{it} \sim N(0;{\sigma }_{e}^{2});{u}_{i} \sim {N}^{+}(\mu ;{\sigma }_{{u}_{i}}^{2}),{u}_{it} \sim {N}^{+}(\lambda ,{\sigma }_{{u}_{it}}^{2}),{b}_{i} \sim N(0;{\sigma }_{b}^{2})$. The deterministic component given by the terms ${\beta }_{0}+{{{{\boldsymbol{x}}}}}_{it}^{{\prime} }{{{\boldsymbol{\beta }}}}$ is the production function mapping the inputs transformed by each airline to move passengers (in our baseline model), with the level of passenger traffic affected also by the shifter. The components *u*_*it*_ have expected values $\lambda =\sqrt{\frac{2}{\pi }{\sigma }_{{u}_{it}}^{2}}$ that depend on a set of variables (exogenous determinants of the transient inefficiency) through the linear model:

2 $$\log \left({\sigma }_{{u}_{it}}^{2}\right)={\gamma }_{0}+{{{{\boldsymbol{z}}}}}_{it}^{{\prime} }{{{\boldsymbol{\gamma }}}},$$

where $\log ({\sigma }_{{u}_{it}}^{2})$ is the logarithm of the transient inefficiency variance, ${{{{\boldsymbol{z}}}}}_{it}^{{\prime} }$ is a row vector of *q* exogenous determinants of transient inefficiency and ***γ*** is a column vector of *q* unknown parameters. Moreover, the persistent inefficiency components *u*_*i*_ have expected value ${\mu }_{i}=\sqrt{\frac{2}{\pi }{\sigma }_{{u}_{i}}^{2}}$ that depends on exogenous determinants through the following linear model:

3 $$\log \left({\sigma }_{{u}_{i}}^{2}\right)={\delta }_{0}+{{{{\boldsymbol{w}}}}}_{i}^{{\prime} }{{{\boldsymbol{\delta }}}},$$

where $\log ({\sigma }_{{u}_{i}}^{2})$ is the logarithm of the persistent inefficiency variance, ${{{{\boldsymbol{w}}}}}_{i}^{{\prime} }$ is a row vector of ${q}^{{\prime} }$ exogenous determinants of persistent inefficiency and ***δ*** a column vector of ${q}^{{\prime} }$ parameters.^3^

We fit model (1) with the additional equations (2)–(3) for the determinants of efficiencies under two functional specifications (if convergence in ML estimation is achieved, that might not occur if the number of independent variables is too large): (1) Cobb-Douglas; (2) translog.^4^ The equation representing the translog airline production function is:

4 $$\ln ({y}_{it})={\beta }_{0}+\mathop{\sum }\limits_{k=1}^{p}{\beta }_{k}\ln ({x}_{it})+\frac{1}{2}\mathop{\sum }\limits_{k=1}^{p}\mathop{\sum }\limits_{j=1}^{p}{\beta }_{kj}\ln ({x}_{kit})\ln ({x}_{jit})+\theta {f}_{it}+{b}_{i}-{u}_{it}-{u}_{i}+{e}_{it}$$

where *β*_*kj*_ = *β*_*jk*_. The translog production function collapses to the Cobb-Douglas production function if *β*_*kj*_ = 0, *j* = 1, 2, …, *p*, *k* = 1, 2, 3, …*p*. One of the main assumptions of the model (1) is that unobserved heterogeneity is uncorrelated with the frontier regressors. In order to have control over this assumption, we implement the Mundlak (1978) approach. We add to Eq. (4) the means over time of the time-varying input variables: ${\overline{x}}_{i}=\frac{1}{T}\mathop{\sum }\nolimits_{t = 1}^{T}\ln {x}_{it}$, so that we can rewrite Eq. (4) as follows:

5 $$\begin{array}{rcl}\ln ({y}_{it})&=&{\beta }_{0}+\mathop{\sum }\limits_{k=1}^{p}{\beta }_{k}\ln ({x}_{it})+\frac{1}{2}\mathop{\sum }\limits_{k=1}^{p}\mathop{\sum }\limits_{j=1}^{p}{\beta }_{kj}\ln ({x}_{kit})\ln ({x}_{jit})\\ &&+\mathop{\sum }\limits_{k=1}^{p}{\eta }_{k}{\overline{x}}_{ki}+\theta {f}_{it}+{b}_{i}-{u}_{it}-{u}_{i}+{e}_{it}\end{array}$$

We test the joint significance of the Mundlak terms on the basis of a likelihood ratio test. The Cobb-Douglas production function has output-input elasticities given by the first-order coefficients, i.e., *ϵ*_*y*,*k*_ = *β*_*k*_. In the translog production function, these elasticities depend instead on the level of the inputs, i.e., ${\epsilon }_{y,k}={\beta }_{k}+\mathop{\sum }\nolimits_{j = 1}^{p}{\beta }_{kj}\ln ({x}_{jit})$.

Other popular SF models for panel data are nested in the model (1). For instance, the time-invariant Pitt and Lee (1981) model is obtained by dropping the random components *u*_*it*_, and *b*_*i*_ from model (1). Since Colombi et al. (2014) persistent and transient inefficiency SF model is based on random components, we will compare its estimates with those obtained with a true random effect (TRE) SF model (Greene 2005a, b), which is obtained by dropping the random term *u*_*i*_ from model (1).

As already anticipated, in our baseline framework each airline uses two inputs, labor, and capital; labor is given by the annual number of employees (pilots, flight attendances, ground, and others); capital is related to maximum passenger transport capacity, i.e., the seats available in the airline’s fleet. This measure of capital incorporates the size of the aircraft in the airline fleet, information that is instead ignored by contributions using simply the number of planes. Regarding the possible determinants of the two inefficiency terms in (2)–(3), we investigate the impact on airlines efficiency of the following factors: airline public ownership, the political stability of the country where the airline headquarters is located, and whether the airline’s headquarter is located in a sub-Saharan country. Public ownership (*PUB*) is a dummy variable equal to 1 if the local government has more than 50% of the airline shares: in this case, we classify the airline as one with public ownership. Despite general evidence suggesting an association between public ownership and inefficiency, the implication of government control are not clear a priori in a context like the African aviation market. Indeed, government involvement may favour stability in a volatile and not fully liberalized market environment, supporting travel demand.

The country’s level of political stability (*POLSTAB*) is a continuous variable given by a World Bank index (see Section 5 for details). Africa is a continent where *coups d’état* frequently occur, and where political systems are often very fragile, not guaranteeing stability for government formations. Political stability and economic growth are generally considered as interconnected factors (Dalyop 2019). However, political stability is not necessarily good, especially in less developed countries, since it may give rise to an entrenched government where incentives towards efficiency are rather low, in favor of different forms of opportunistic behavior (Hussain 2014).^5^

*SUBSAHARA* is a dummy variable equal to one if the reference country for the airline is located South of the Sahara desert. Sub-Saharan countries differ from Egypt, Libya, Algeria, Tunisia, and Morocco, which are on the Mediterranean sea, and benefit from greater possibilities of exchange with the European countries, especially those of Southern Europe. This could have an effect on airlines in this African region seeking efficiency. Button et al. (2022) confirm that there is an important geographical separation in air transportation between Mediterranean countries and those South of the Sahara desert, many of which are landlocked.

The econometric model (1) of the production frontier and the efficiency of African airlines is estimated with ML as follows:

6 $$\begin{array}{lll}\log {(PAX)}_{it}={\beta }_{0}+{\beta }_{1}\times \log {(K)}_{it}+{\beta }_{2}\times \log {(L)}_{it}\\ \qquad\qquad\qquad\quad+\,\theta \times BETWEE{N}_{it}+{b}_{i}-{u}_{it}-{u}_{i}+{e}_{it}\end{array}$$

7 $$\begin{array}{ll}\log ({\sigma }_{{u}_{i}})={\delta }_{0}+{\delta }_{1}\times PU{B}_{it}+{\delta }_{2}\\ \qquad\qquad\quad\times\, POLSTA{B}_{it}+{\delta }_{3}\times SUBSAHAR{A}_{i}\end{array}$$

8 $$\begin{array}{ll}\log ({\sigma }_{{u}_{it}})={\gamma }_{0}+{\gamma }_{1}\times PU{B}_{it}+{\gamma }_{2}\\ \qquad\qquad\quad\times \,POLSTA{B}_{it}+{\gamma }_{3}\times SUBSAHAR{A}_{i}\end{array}$$

where (6) is the Cobb-Douglas production frontier, which can be augmented by including the Mundlax correction terms $\overline{\log {(K)}_{it}},\overline{\log {(L)}_{it}}$, and by adding the quadratic and interaction terms for the translog specification (i.e., ${(\log {(K)}_{it})}^{2}$), ${(\log {(L)}_{it})}^{2}\left.\right),\log {(K)}_{it}\times \log {(L)}_{it}$).

### Alternative model specifications

In this Section the alternative specifications of our model are presented. First, a different output variable is considered in the analysis, i.e., revenue per passenger kilometers (*RPK*). RPK is a typical measure of traffic used in the aviation industry and is computed as the sum of the product between the number of revenue-paying passengers on each flight and the distance flown. As a result, RPK allows to take into account not only the passenger volumes, but also the distance flown. Flying longer distances implies that, *ceteris paribus*, more of the inputs are used, especially fuel. Therefore, using RPK as an alternative output measure requires, for consistency, the inclusion of fuel consumption (*F*) among the input variables of the production process. Hence, the second main novelty consists in the inclusion of fuel consumption as an additional input variable. This is in line with many previous studies on airline technical efficiency (Scotti and Volta 2017, 2018). How fuel consumption is proxied is explained in Section 5.

The above mentioned changes transform our production function in Eq. (9):

9 $$\begin{array}{ll}\log {(RPK)}_{it}={\beta }_{0}+{\beta }_{1}\times \log {(K)}_{it}+{\beta }_{2}\times \log {(L)}_{it}+{\beta }_{3}\\ \qquad\qquad\qquad\quad\times \log {(F)}_{it}+\theta \times BETWEE{N}_{it}+{b}_{i}-{u}_{it}-{u}_{i}+{e}_{it}\end{array}$$

As discussed extensively in Section 8, we note that the inclusion of fuel consumption, which certainly is of interest for the analysis, can be done only at the cost of renouncing flexible functional forms (i.e., the translog in our case) because the number of parameters to be estimated increases significantly in relation to the limited size of the sample.

The other major change made to our base model regards the determinants of the two inefficiency terms in (2)–(3). Two additional factors are incorporated in the analysis, i.e., the degree of the country’s economic freedom and the membership to one of the three global airline alliances.

The country’s level of Economic Freedom provides insights about the overall economic environment as the level of government intervention in the economy, the strength of property rights, the level of corruption, and the effectiveness of the legal system, among others. In Africa, some countries in the region have made progress in reducing government intervention, improving property rights, and tackling corruption, but many still face significant challenges in these areas. More details on the variable characteristics are provided in Section 5. By introducing a measure of economic freedom our purpose is to get insight into the effect of openness to competition that may, in turn, indicate the implication of fostering deregulation in the African aviation markets.

Also alliance membership can play a role given the potential consequent operational benefits discussed in Section 3 (e.g., better optimization of the schedules and improved efficiency of the networks). Indeed, Button et al. (2022) provide evidence of the positive impact of joining an alliance both in terms of passengers and load factors for African carriers.

These determinants of inefficiencies are tested in different combinations taking into account, on the one hand, the willingness to grasp some additional insights on the drivers of efficiency in the African airline industry and, on the other hand, the already mentioned greater difficulties in achieving convergence in the estimation. Again, the limitations of these alternative specifications are discussed in Section 8. More specifically, we estimate (i) a model with the same efficiency determinants of our base model, i.e., *PUB*, *POLSTAB*, and *SUBSAHARA*, (ii) a model with only *ECONFREE*, (iii) a model with only *ALLIANCE*, (iv) a model with *PUB* and *ALLIANCE*, (v) a model with *PUB* and *ECONFREE*, and (vi) a model with *POLSTAB* and *ECONFREE*.

## The data

Data on the African aviation market are less comprehensive than the information available for the US, Europe, and Asian markets. To estimate the model presented in Eq. (1) we build a new data set regarding carriers members of the African Airlines Association (AFRAA) for the period 2010–19, i.e., 10 years.^6^ The data set relates to the major African airlines and is constructed from different sources. Much of the data used here are taken from the AFRAA annual reports and integrated with other official and website sources.^7^

The data mining process results in a balanced panel data set including airline-year data of 17 major African carriers in 10 years (i.e., 170 observations). We download all the annual reports released by AFRAA from 2011 to 2020 in order to get the number of passengers, employees, ownership, and fleet details.^8^ Then we matched the fleet with the capacity of each aircraft from OAG to express the size of the fleet (*K*_*it*_) in terms of available seats. This measure provides a better estimate than simply counting the number of aircraft because it takes into account also their size.

The betweenness centralization variable (*BETWEEN*) is an index capturing the airline network structure. Network measures are important indicators to describe the characteristics of air networks and are currently used in different contributions (e.g., Ciliberto et al. 2019, Roucolle et al. 2020). In particular, centralization is a measure at the network level that is built by aggregating in a unique index the centrality measures of all the nodes (airports) of an airline network. While centrality captures the importance of a single airport in a network, centralization measures how much important is the most relevant airport compared to all the other airports belonging to the same network - namely how much a given air network is centralized in its most relevant airport. More specifically, in the case of betweenness, an airport centrality is greater the higher the proportion of shortest routes between pairs of airports on which the airport of interest acts as an intermediate stop. For an airport *a* in the network of airline *i*, betweenness centrality at time $t,{C}_{at}^{i}$, is computed as shown in Eq. (10)

10 $${C}_{at}^{i}=\mathop{\sum }\limits_{j\ne a\ne k}^{}\frac{{\psi }_{jk}^{a}}{{\psi }_{jk}}$$

where *ψ*^*a*^ is the number of shortest paths between airports *j* and *k* on which *a* acts as an intermediate stop; *ψ*_*jk*_ is the total number of shortest paths between *j* and *k*. A network betweenness centralization (*BETWEEN*) measures, as anticipated in Section 4, how close is a specific network to a pure (star) H&S and it is computed as shown in Eq. (11), where the numerator is the sum of the differences between the betweenness centrality of the most central airport in airline *i*’s network (${C}_{at}^{i* }$) and the betweenness centrality of all the other airports in the network, while the denominator is the maximum theoretical value of such difference in a network with *N* nodes, namely the one of a H&S network where the hub airport operates as the center of a star, and all the spokes are directly connected to it.^9^

11 $$BETWEE{N}_{it}=\frac{\mathop{\sum }\nolimits_{i = 1}^{N}{C}_{at}^{i* }-{C}_{at}^{i}}{\max{\mathop{\sum }\nolimits_{i = 1}^{N}{C}_{at}^{i* }-{C}_{at}^{i}}}$$

*POLSTAB*_*it*_ is the World Bank indicator that indicates Political Stability and Absence of Violence/Terrorism and measures perceptions of the likelihood of political instability and/or politically motivated violence, including terrorism. The variable is re-scaled to be positive and greater than one, since it is subject to a logarithmic transformation. *PUB*_*it*_ is a dummy variable that takes value one if the majority of the ownership is public, and 0 otherwise. *SUBSAHARA*_*i*_ is a dummy variable equal to 1 if the carrier is located in a Sub-Saharan country, and 0 otherwise.

Table 1 presents the descriptive statistics of our sample. According to AFRAA, on average African carriers moved 2.8 million passengers per year (*PAX*_*it*_), ranging from 46,851 carried by *Asky Airlines* in 2011 to almost 13 million by *Ethiopian Airlines* in 2019. The standard deviation higher than the mean indicates that there is a relevant variation in size among African carriers. The representative airline fleet consists of 4,592 seats (*K*_*it*_), with a minimum of 185 seats and a maximum of 23,855. Average employment is 4,148 people (*L*_*it*_); again standard deviation is rather high, the minimum is only 157 employees, and the maximum is 32,805. In the estimates, *PAX*_*it*_, *K*_*it*_, and *L*_*it*_ are mean scaled using the geometric mean to standardize the variables and reduce the impact of possible outliers.

**Table 1:** Descriptive statistics

Variable	Mean	S.d.	Min	Max	Unit	Description
*PAX*_*it*_	2772	2907	47	12,631	000	Annual passengers
*K*_*it*_	4592	4951	185	23,855	number	Available seats fleet size
*L*_*it*_	4148	6800	157	32,805	number	Flight and ground personnel
*BETWEEN*_*it*_	0.83	0.18	0.34	1	index	Centralization of airline network
*PUB*_*it*_	0.82		0	1	dummy	Public control
*POLSTAB*_*it*_	3.00	0.79	1.62	4.41	index	Country political stability
*SUBSAHARA*_*i*_	0.76		0	1	dummy	Sub-Saharan country

Betweenness centralization (*BETWEEN*_*it*_) has an average equal to 0.83, it ranges from 0.34 of *LAM Mozambique* to 1 of *Air Seychelles*.^10^*POLSTAB*_*it*_ has a mean equal to 3, with a minimum equal to 1.62 (Ethiopia) and a maximum equal to 4.41 (Botswana). In our sample, 82% of observations are related to a public ownership airline (*PUB*_*it*_). About 76% of African airlines in our sample are located in sub-Saharan Africa.

### Additional variables for alternative specifications

We now discuss the definitions and the descriptive statistics of the set of additional variables included in the alternative specifications of our baseline model, i.e., model (1). As explained in Section 4, these variables are revenue-passenger-kilometers (*RPK*), fuel consumption (*F*), countries’ economic freedom (*ECONFREE*), and alliance membership (*ALLIANCE*).

RPK is sourced from AFRAA reports, while aircraft fuel consumption is difficult to compute because it depends on several factors (e.g., aircraft and engine models, distance flown, load factor). In the case of African airlines, it is even harder to collect all the necessary information given the already-mentioned data scarcity. Our approach consists of computing a proxy of airline fuel consumption starting from the available information. More specifically, the airline’s yearly fuel consumption is computed on the basis of three parameters, i.e., (1) the age of the fleet, (2) the distance flown, and (3) the number of passengers. OAG provides the aircraft model and the distance flown associated with each flight. Furthermore, for each aircraft model *m* we identify the age as the difference between the year of observation and the year of the first flight of that model.^11^ Then, we compute a “relative” fuel consumption in gallons according to the following equation: ${F}_{it}=\mathop{\sum }\nolimits_{a\in i}^{A}\frac{SEAT{S}_{mit}\times DISTANC{E}_{mit}\times {(1+0.0288)}^{AG{E}_{mt}}}{100}$, where 0.0288 is an annual penalization for each additional age year. Indeed, Chèze et al. (2011) show that energy efficiency improvements have been equal to 2.88% per year during the 1983–2006 period. The fraction *SEATS*/100 is taken from OpenAirlines (2022), stating that fuel consumption in commercial air transportation is around 1 gallon of kerosene per passenger per 100 kilometers flown.^12^ In this case, we use seats as a proxy for passengers.

The country’s level of economic freedom is a continuous variable measured by the Heritage Foundation Economic Freedom Index. It assesses the degree of economic freedom in each country based on 12 quantitative and qualitative factors grouped into four broad categories: (i) rule of law, (ii) government size, (iv) regulatory efficiency, and (iv) open markets. These components are used to assign a score on a scale between 0 and 100, with 100 representing the highest level of economic freedom. It provides insights into the overall economic environment of the country that may impact the African air carriers. In Africa, some countries in the region have made progress in reducing government intervention, improving property rights, and tackling corruption, but many still face significant challenges in these areas.

*ALLIANCE* is a dichotomous variable that represents the status of an airline as a full member of one of the three main global alliances. The variable takes the value of 1 if the carrier is an alliance member, and 0 otherwise. If a carrier joins an alliance in a given year, the variable is equal to 1 the following year, as it takes time for the carrier to become a full member and fully integrate into the alliance network. This is the case of *Ethiopian Airlines* and *Kenya Airways* that joined *Star Alliance* and *Skyteam* respectively in December 2011 and June 2010. Table 2 presents the descriptive statistics of the additional variables.

**Table 2:** Descriptive statistics for variables used in alternative specifications

Variable	Mean	S.d.	Min	Max	Unit	Description
*RPK*_*it*_	6,960,037	8,508,729	103,261	45,289,320	000	# of annual RPK
*FUEL*_*it*_	184,799,015	223,395,655	3,319,606	1,134,108,806	number	Gallons of fuel consumed
*ALLIANCE*_*it*_	0.22		0	1	dummy	Member of an alliance
*ECONFREE*_*it*_	58.4	7.5	44.7	77	index	Country economic freedom

On average African carriers moved 6.9 billion RPK per year (*RPK*_*it*_), ranging from 103 million to about 45 billion in 2019. The standard deviation is higher than the mean and this confirms the high dispersion in passenger volumes among African carriers. The fuel consumption of the representative airline fleet consists of about 185 million gallons, while only 22% of the observations in our sample are related to members of a global alliance. The average index of economic freedom is 58.4, with a small standard deviation, and a maximum equal to 77, quite far from 100, indicating full economic freedom.

## Empirical results

The estimates of African airlines’ production frontier and determinants of inefficiency in our baseline model shown in Eqn. (6)–(8) are reported in Table 3, which is split into two parts. The top rows display the estimated coefficients of the inputs *K* and *L* and of the production shifter (*BETWEEN*). The heading, in this case, is given by the dependent variable of the baseline model, i.e., $\log (PAX)$. The bottom rows show instead the estimated coefficients of the factors affecting inefficiency. Columns (1)–(4) present the results of Greene (2005a, b) TRE model. In this case, only time-varying inefficiency is included, and the estimated coefficients of *PUB*, *POLSTAB*, *SUBSAHARA* are reported at the bottom rows of Table 3. Columns (5)–(8) show the estimates of Colombi et al. (2014, 2017) four random components SF model, that considers both time-varying and time-invariant inefficiencies. The impacts of the factors affecting time-invariant inefficiency are displayed above those related to time-varying inefficiencies. Table 3 presents estimates both for the Cobb-Douglas production function (columns (1)–(2), and (5)–(6)) and for the translog one (columns (3)–(4), and (7)–(8)). The difference in each pair of columns is given by the inclusion in the estimated model of the Mundlak correction variables ($\overline{\log (K)},\overline{\log (L)}$). The likelihood-ratio test shows that the Mundlak correction variables are an important improvement in the model fit under the translog functional form (the statistics are 19.1, and the *p*-value is 0.0001), but not for the Cobb-Douglas specification.

**Table 3:** African airlines production frontier and determinants of transient and permanent inefficiency

	Dependent variable: $\log (PAX)$							
Independent variables	(1)	(2)	(3)	(4)	(5)	(6)	(7)	(8)
$\log (K)$	0.6184***	0.5906***	0.5542***	0.4960***	0.6332***	0.5275***	0.6241***	0.5056***
	(0.0501)	(0.0733)	(0.0593)	(0.0536)	(0.0147)	(0.0531)	(0.0188)	(0.0522)
$\log (L)$	0.1795***	0.1423*	0.2458***	0.0971′	0.1723***	0.1408***	0.1325***	0.0676***
	(0.0404)	(0.0602)	(0.0418)	(0.0521)	(0.0249)	(0.0425)	(0.0701)	(0.0185)
*BETWEEN*	0.1929	0.1670	0.1611	0.1911′	0.1831***	0.1688	0.2012	0.2082***
	(0.1544)	(0.1503)	(0.1383)	(0.1109)	(0.0311)	(0.1075)	(0.2567)	(0.0560)
$\overline{\log (K)}$		0.0099		0.0974		0.1513***		0.0806***
		(0.0996)		(0.0753)		(0.0698)		(0.0210)
$\overline{\log (L)}$		0.0840		0.2517***		0.0609		0.2203***
		(0.0798)		(0.0720)		(0.0457)		(0.0277)
${\left(\log (K)\right)}^{2}$			0.0521	0.1251			0.0712	0.0264
			(0.1162)	(0.0914)			(0.0570)	(0.0256)
${(\log (L))}^{2}$			−0.0860	−0.2178***			−0.0490′	−0.2045***
			(0.1024)	(0.0747)			(0.0256)	(0.0295)
$\log (K)\times \log (L)$			0.0746	0.0119			−0.0714)	0.0871***
			(0.1906)	(0.1624)			(0.0547)	(0.0210)
Constant	0.0625	0.0842	0.0629	0.2716**	0.1343***	0.1846***	0.6470	0.6029***
	(0.1433)	(0.1267)	(0.1383)	(0.1049)	(0.0233)	(0.0348)	(0.1571)	(0.0474)

	Factors affecting inefficiency							
	Time invariant inefficiency							
Determinants of inefficiency								
*PUB*					−5.7111***	−2.0045***	−2.0844***	−1.5571***
					(0.0210)	(0.0581)	(0.0663)	(0.0224)
*POLSTAB*					4.9012***	1.5376***	1.9592***	1.4785***
					(0.0285)	(0.1427)	(0.1524)	(0.0342)
*SUBSAHARA*					−5.5309***	−3.3612***	2.3659***	1.4037***
					(0.0247)	(0.1089)	(0.0585)	(0.0463)
Constant					−14.8287***	−14.1103***	−3.5270***	−2.8551***
					(0.0404)	(0.0605)	(0.1569)	(0.0456)

	Time-varying inefficiency							
*PUB*	−1.3531***	−1.3199***	−1.2298***	−0.8132*	−1.3291***	−1.1088***	−0.9654***	−0.8918***
	(0.3903)	(0.3780)	(0.3949)	(0.3223)	(0.0388)	(0.1292)	(0.1115)	(0.0360)
*POLSTAB*	0.5786	0.2889	0.6885	−0.3701	0.4828***	−0.5984***	−0.7775***	−1.6218***
	(0.8457)	(0.8588)	(1.1045)	(0.5784)	(0.0192)	(0.0873)	(0.2202)	(0.0415)
*SUBSAHARA*	32.1002	35.9882	1.6258′	1.9565***	4.1870***	1.7461***	1.8896***	2.3723***
	(676.9505)	(1178.701)	(0.9111)	(0.3850)	(0.0141)	(0.0725)	(0.0214)	(0.0168)
Constant	−33.7183	−37.2849	−3.4391*	−2.5153***	−2.0726***	0.3770***	4.1430***	−5.8795***
	(676.9487)	(1178.702)	(1.6796)	(0.6110)	(0.0292)	(0.0370)	(0.1761)	(0.0333)
log-likelihood	1.0426	1.7039	−0.7545	8.7949	−0.2585	2.2373	4.7007	6.8082

variables with overbar are Mundlak correction

***0.1% significance level, **1% significance level, *5% significance level, ′10% significance level

The results on the deterministic part of the estimated translog SF models (column (8)) confirm that the four-random components approach provides better results. The estimated coefficients are almost all statistically significant. First-order input coefficients $\log K,\log L$ are both positive and statistically significant, as well as those related to the Mundlak correction variables $\overline{\log K},\overline{\log L}$. The second-order estimated coefficient for labor input is negative (-0.2045) and significant, while that of the input interaction variable $\log K\times \log L$ is positive (0.0871) and significant. First-order input coefficients are positive and significant also with the TRE Cobb-Douglas model (columns (1)–(2)), with the TRE translog model (columns (3)–(4), weakly significant $\log L$ in column (4)), with the Cobb-Douglas four random components model (columns (5)–(6)), and with the four random components model without Mundlak corrections.

Interestingly, the estimated coefficient of the production function shifter *BETWEEN* is positive and significant in the baseline model only with the Colombi et al. (2014, 2017) SFA (column (8)), and equal to +0.2082. This confirms that airlines with higher network centralization are associated with higher passenger traffic. A positive estimated coefficient for the network centralization is also obtained with the four-random component SFA in the case of the Cobb-Douglas production function without Mundlak corrections.

By splitting airlines’ inefficiency into persistent (*u*_*i*_) and transient (*u*_*it*_), and by separating unobserved heterogeneity from them, we obtain better results also regarding the factors affecting the different inefficiency types, as shown in columns (5)–(8) of Table 3, bottom rows. The TRE model identifies only one determinant, i.e., if the airline has public ownership the transient inefficiency is lower. Limited to the translog production function with Mundlak corrections, the TRE model also identifies sub-Sahara as a negative determinant of transient efficiency.

The four random component model identifies instead the following results. Public ownership (*PUB*) always decreases inefficiency, both persistent and transient. This finding confirms previous evidence on African airlines, differently from what is observed in more developed air transport markets, where there is more evidence that private airlines are more efficient (e.g., Yu et al. (2019)). Our evidence is even stronger than that provided in the existing literature because it shows that both persistent and transient efficiencies are positively affected by public ownership. However, this result must be interpreted carefully, especially from a policy perspective. In line with Barros and Wanke (2015), we believe that this condition is linked to protectionism.

We also find that higher political stability (*POLSTAB*) improves time-varying inefficiency, while it increases persistent inefficiency. This result may appear surprising in a way. As pointed out by Colombi et al. (2017), persistent inefficiency is mainly due to long-run moral hazard, e.g., obsolete equipment that is not substituted. In air transportation may be due to an aircraft fleet in excess compared to the demand, for which often the available seats are in excess, leading to low load factors; or too many personnel. Since political stability is an indicator linked to the absence of government violent overturns, and since it is not rare in Africa the presence of political power concentrated in the hands of a single person, and for a long time, this may lead to political interference in airline employment levels and lower incentives in the efficient use of capital. In this sense, *POLSTAB* may capture a negative side of government control (and protectionism) in terms of long-run effect on efficiency. On the contrary political stability decreases short-run inefficiency, providing incentives to limit short-run moral hazard behavior, e.g., inefficient supplier selection and sub-optimal resource allocation, or trial-and-error processes in unknown situations. Another possible interpretation may be connected to political practices aimed at “buying political stability in the short run”, but not necessarily favorable to the development in the long run of the economy and consequently also of the airline industry. However, the evidence on *POLSTAB* has to be interpreted carefully and further arguments will be provided when we will discuss the evidence obtained with the alternative specifications of our baseline model.

Last, column (8) of Table 3 shows that airlines located in sub-Saharan countries have both higher persistent and transient inefficiencies. As expected, airlines closer to Europe benefit from higher influences and transactions with European countries, they are also operating under an open sky agreement (e.g., Morocco and Tunisia signed agreements under the European Neighborhood Policy, that aims to increase economic integration between European Union members and surrounding countries; Bernardo and Fageda 2017), and this higher level of competition provides incentives toward lower inefficiency levels.

**Fig. 1:**
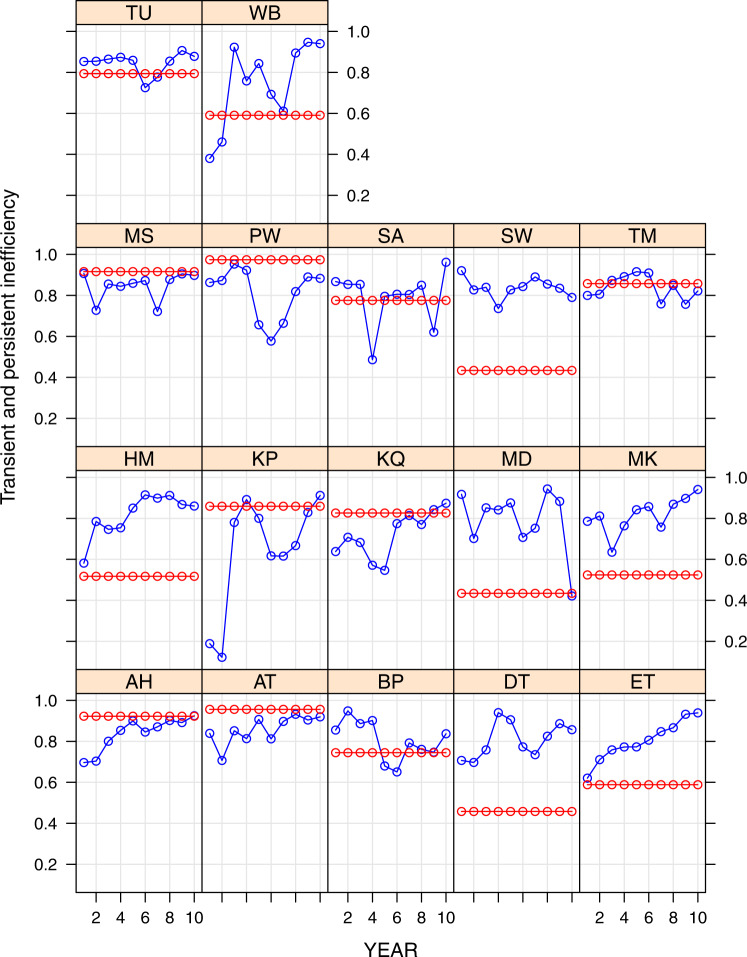
Persistent and transient efficiency in African airlines

From the estimated frontier we can compute the efficiency scores of each African airline. More specifically, we calculate the efficiency scores according to Colombi et al. (2014) from the estimated coefficients shown in column (8) of Table 3. Figure 1 shows the details and the dynamics of each airline’s efficiency scores during the period 2010-2019, separated by persistent (red) and transient (blu) efficiency.

Some interesting insights are derived from the analysis of efficiency scores. At least four African airlines (i.e., *Egyptair* (MS), *Precision Air* (PW), *Air Algérie* (AH), and *Royal Air Maroc* (AT)) have persistent efficiency always higher than transient efficiency, while six airlines have persistent efficiency always lower than transient efficiency (i.e., *Air Namibia* (SW), *Air Seychelles* (HM), *Air Madagascar* (MD), *Air Mauritius* (MK), *Taag Angola* (DT), and *Ethiopian Airlines* (ET)). The other 7 airlines have years where persistent efficiency is higher than transient one, and vice-versa. Regarding transient efficiency, some airlines are improving it during the observed period, as shown by an upward trend in Fig. 1: *RwandAir* (WB), *Air Seychelles* (HM), *Asky Airline* (KP), *Kenya Airways* (KQ), *Air Mauritius* (MK), *Air Algérie* (AH), *Royal Air Maroc* (AT), and *Ethiopian Airlines* (ET). *Air Namibia* (SW), and *Air Madagascar* (MD) have instead decreased their transient efficiency levels over the observed period, while all other African airlines have at the end of the period about the same transient efficiency they had at the beginning.

Table 4 provides the details of the distribution of the efficiency scores by efficiency types and by different levels of the factors affecting airlines’ technical performances. The first two rows of Table 4 show the descriptive statistics of persistent and transient efficiency scores: the latter has a mean equal to 80%, higher than the mean of the former (72%). Transient efficiency, on average, is about 73% at the beginning of the observed decade (2010), goes up to 85% in 2017, and stays more or less at that level until the end of the period (0.86% in 2019). These are of course relative levels of efficiency, but indicate that diffused technical inefficiency is an issue that should be addressed by proper policy interventions in the industry.

**Table 4:** African airlines’ efficiency scores by efficiency types and exogenous factors in the baseline model

Efficiency	Min	1st quartile	Median	Mean	3rd quartile	Max
Persistent	0.43	0.52	0.78	0.72	0.86	0.97
Transient	0.12	0.77	0.84	0.80	0.89	0.96
Dynamics of transient average efficiency						
2010	2011	2012	2015	2017	2018	2019
0.73	0.72	0.82	0.78	0.85	0.86	0.86
Number of persistent efficiency scores in different categories						
	Public	Private				
Inefficient	80 (46%)	0 (0%)				
Moderate	30 (18%)	10 (6%)				
Efficient	30 (18%)	20 (12%)				
	Low *POLSTAB*	High *POLSTAB*				
Inefficient	36 (21%)	44 (26.5%)				
Moderate	36 (21%)	4 (2.5%)				
Efficient	48 (28%)	2 (1%)				
	*SUBSAHARA* = 0	*SUBSAHARA* = 1				
Inefficient	0 (0%)	80 (46%)				
Moderate	10 (6%)	30 (18%)				
Efficient	30 (18%)	20 (12%)				
Number of transient efficiency scores in different categories						
	Public	Private				
Inefficient	65 (38%)	20 (12%)				
Moderate	37 (22%)	5 (3%)				
Efficient	38 (22%)	5 (3%)				
	Low *POLSTAB*	High *POLSTAB*				
Inefficient	60 (35%)	25 (15%)				
Moderate	31 (18%)	11 (7%)				
Efficient	29 (17%)	14 (8%)				
	*SUBSAHARA* = 0	*SUBSAHARA* = 1				
Inefficient	11 (7%)	74 (44%)				
Moderate	16 (9%)	26 (14%)				
Efficient	13 (8%)	30 (18%)				

Based on the 1*st* and 3*rd* quartiles of the distribution we identify three categories of airlines’ technical performances: inefficient airlines if the score is lower than the median, moderately inefficient airlines if the efficiency score is between the median and the 3*rd* quartile, and efficient airlines if the score is higher than the 3*rd* quartile. Regarding persistent efficiency, out of 170 observations, 46% are in the inefficient group and are related to public airlines, while 18% are in the efficient group and are also with public ownership. Private airlines have more observations in the inefficient group. Observations where countries with low political stability (the raw index is below 0) are associated with airlines belonging to the inefficient group are 21% of the sample, while 21% and 28% of observations combine low stable countries to moderately efficient airlines and efficient airlines respectively. Observations of countries with high political stability and with airlines in the inefficient group are 26.5% of the sample, while only 2% and 1% exhibit low political stability combined with airlines respectively in the moderate and efficient categories. Observations of sub-Saharan airlines in the inefficient group are 46% of the sample, 18% are those in the moderate category, and only 12% are in the efficient one. Observations of airlines belonging to countries on the Mediterranean sea in the efficient group are 18% of the sample, while those in the moderate category are only 6%.

Regarding transient efficiency, 22% of the observations regard public airlines in the efficient group. The same applies to the moderate category, while private airlines are found mostly inefficient (12% of the sample, against 5% in each of the other two categories). Airlines facing low political stability in the inefficient group are 35% of the sample (18% those in the moderate category, and 17% in the efficient one). Observations combining countries with high political stability and airlines in the inefficient group are instead 15% of the sample, 7% those with airlines in the moderate category, and 8% in the efficient one. Last, observations referring to sub-Saharan inefficient airlines represent 44% of the sample, 14% to moderately efficient sub-Saharan carriers, and 18% to efficient ones.

Regarding Fig. 1, it is interesting to analyze whether the variation in transient efficiency may be explained by some variability of its determinants at the airline level.^13^ In Fig. 5 in Appendix, panel (a) presents the variability of *POLSTAB* by airline. In the observed period, there is no variation in *PUB*, as well as in *SUBSAHARA* which is time-invariant by definition. *POLSTAB* improved for some airlines, i.e., *Air Madagascar, MD*, *Ethiopian Airlines, ET*, *Air Botswana, BP*, and *Air Mauritius, MK*. Among these airlines, we find improvements in transient efficiency for ET and MK. A worsening in *POLSTAB* is instead observed for *ASKY Airlines, KP*, *Egyptair, MS*, *Precision Air, PW*, *South African Airways, SA*, *Air Namibia, SW*, *LAM Mozambique Airlines, TM*, and *Tunisair, TU*. Among these airlines, we observed a reduction in transient efficiency for MS, PW, SA, and SW.

## Micro-foundations of estimated production function

In this Section, we check to what extent the estimated production function of African airlines fulfills the well-known properties of Microeconomics production theory. To do this, we use the estimates derived from our baseline model. We also draw some consequences in terms of output elasticities, inputs substitutability, and production scale.

**Fig. 2:**
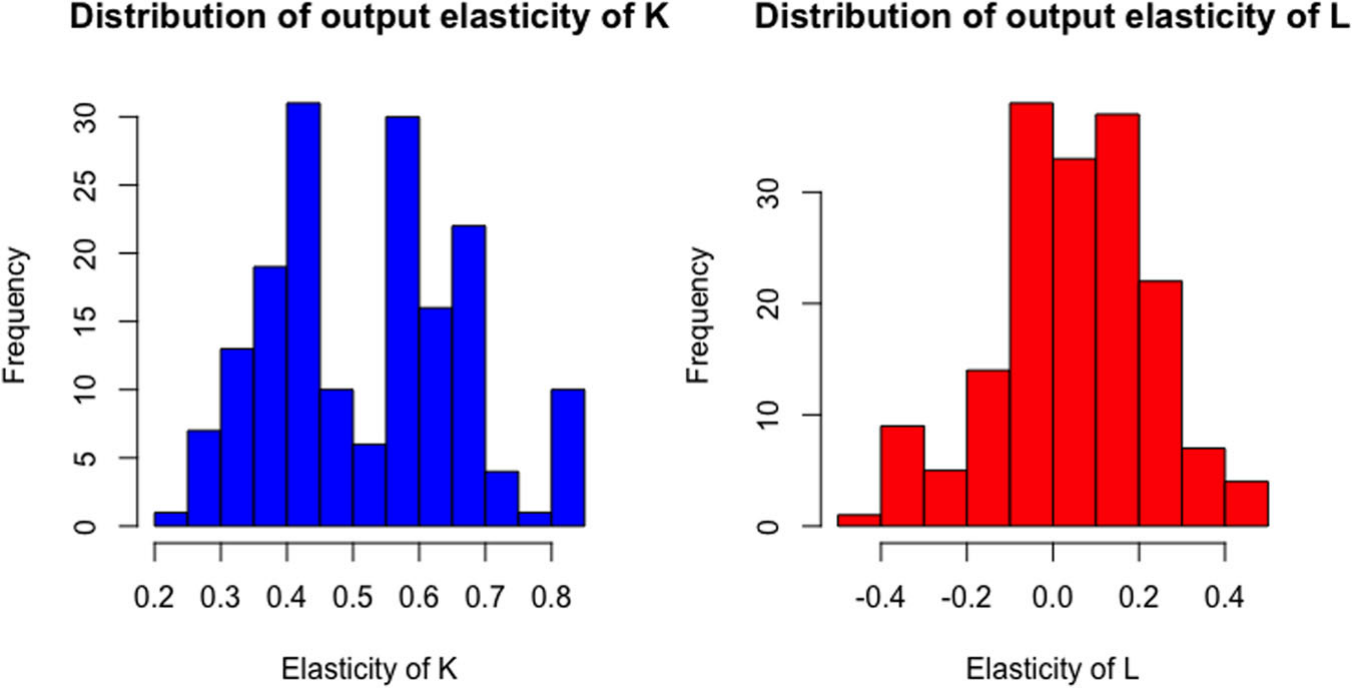
Production function monotonicity condition in African airlines

Fig. 2 presents the distribution of the estimated output elasticities of *K* and *L* for African airlines, using the results from column (8) of Table 3, i.e., the SF model with two inefficiency types and latent heterogeneity. Regarding the output elasticity of capital, all observations fulfill the monotonicity condition between inputs and passengers. As shown in the right panel of Fig. 2, 103 observations out of 170 (61%), have positive output elasticity of labor, while 37% of observations have negative estimates. Regarding input quasi-concavity of the estimated production function, it is necessary to compute the Hessian matrix of second derivatives with respect to *K* and *L*, and check, for each observation, that such matrix is negative semi-definite. A sufficient condition, in this case, is that the principal minor of the Hessian matrix is non-positive and all the following minors alternate in sign. This condition is fulfilled in 154 observations out of 170 (about 91% of the full sample). Hence we may argue that for a rather high proportion of observations, the estimated African airlines’ production frontier shows robust microeconomic foundations.

**Fig. 3:**
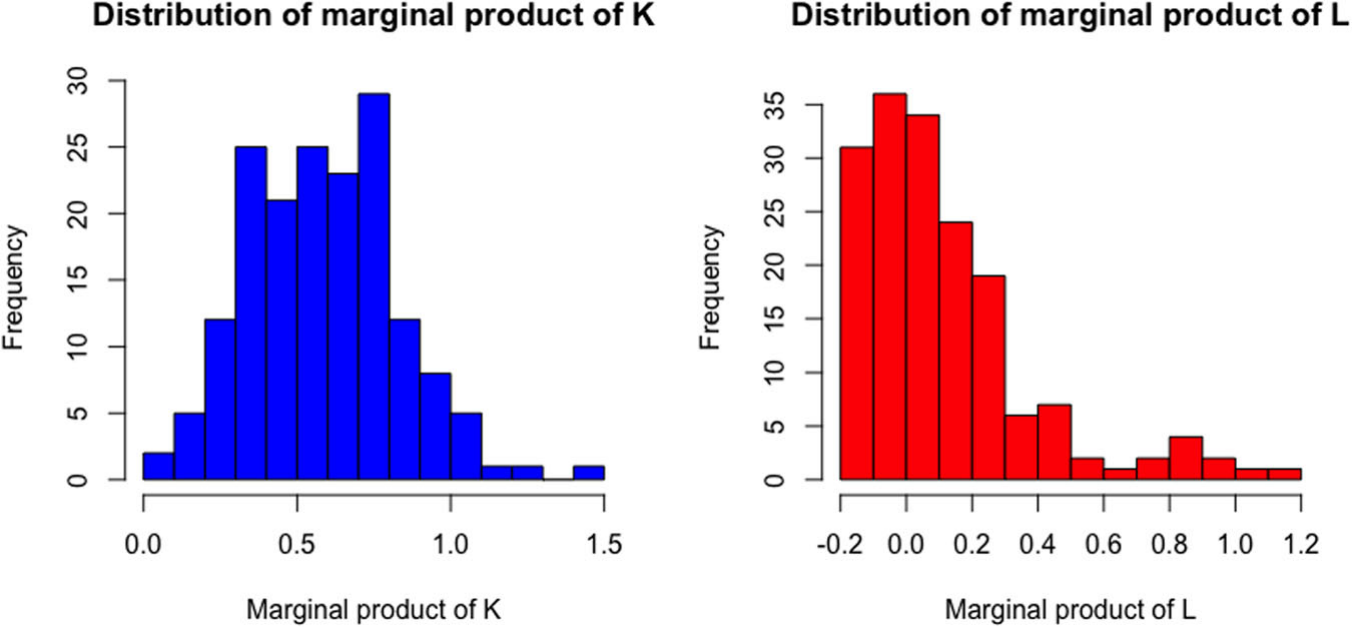
Marginal products of *K* and *L* in African airlines

Being the output and inputs of the estimated production function mean scaled, the first-order estimated coefficients $\log (K),\log (L)$ represent the overall output elasticity of capital and labor respectively. This implies that a +1% in the capital (i.e., fleet capacity) gives rise to +0.51% of passengers, while the same percentage increase in the labor force generates an upward shift in annual passengers equal to +0.07%. Figure 3 displays the distribution of the marginal product of *K* and *L* in African airlines computed for each observation in our sample. While the marginal product of capital is always positive, for some observations we have a negative marginal product of labor (a similar pattern to that observed for output elasticity of labor). This evidence may be explained by the inefficient use of personnel, maybe due to political reasons.

**Fig. 4:**
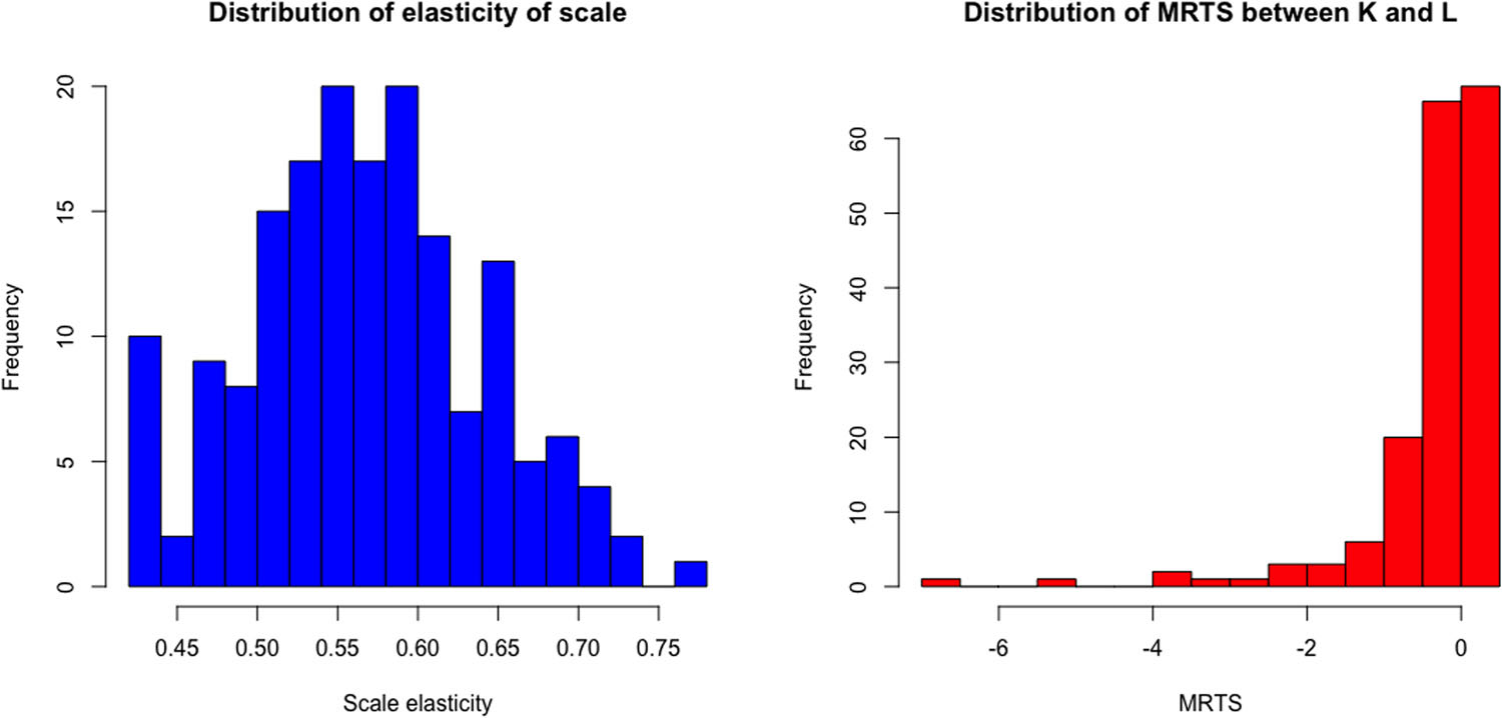
Scale efficiency and *MRTS*_*it*_ in African airlines

Figure 4 left panel presents the distribution of the estimated scale elasticity. On average it is equal to 0.57, and its maximum estimated value is equal to 0.78. This implies that African airlines are operating under decreasing returns to scale, i.e., there is an amount of extra capacity and additional use of labor in this continent’s air transportation sector.

From the estimated production function we can get the marginal rate of technical substitution (*MRTS*_*KL*_) between labor and capital in the African airlines. The right panel of Fig. 4 displays the distribution of the marginal rate of technical substitution between capital and labor in our sample. The average *MRTS*_*KL*_ is equal to −0.39, while the relative *MRTS*_*KL*_ is equal to −0.20. These averages imply that if an airline wants to increase the use of labor by one unit, it has to reduce the use of capital by 0.39 units. The relative marginal rate of technical substitution is the negative ratio between the two output elasticities, in this case with the labor elasticity at the numerator. It means that if an airline aims at increasing labor by 1% it should reduce capital by 0.2%. The estimated *MRTS*_*KL*_ is positive for 103 out of 170 observations (61%). The remaining observations are not efficiently using the inputs, given that they exhibit inputs’ complementarity rather than substitutability.

Last, from the estimated production frontier we compute the direct elasticity of substitution.^14^ The average value in the observed sample is equal to 1.15; hence we observe, in the representative African airline, that capital and labor are substitutes.

## Results from alternative specifications of the African airlines efficiency model

As discussed in Section 4 it is interesting to analyze some alternative specifications of our baseline model of the African airlines’ production function. In this way it is possible, on the one hand, to test whether the results concerning the determinants of inefficiencies of our baseline model are confirmed also in the case of a different output measure and an additional input. On the other hand, the inclusion of new determinants of inefficiencies allows getting more insights into the factors affecting efficiency as well as verifying whether some of the effects previously identified and peculiar to Africa (e.g., the positive impact on the efficiency of public ownership) are robust. In this regard, of particular interest is the role played by the degree of economic freedom of the country where a given airline is based. This factor can highlight how much relevant the development of economies more oriented towards the free market (and therefore less protected by the government umbrella) is to the efficiency of African airlines.^15^

As a first alternative specification we test our previous findings by changing the output variable (i.e., *RPK* in place of *PAX*), and by adding a third input (i.e., fuel consumption *F*). Both variables are expressed in logarithms. Then, keeping *RPK* and *F* in the production function model, we incorporate in the analysis new determinants of inefficiencies, i.e., the degree of economic freedom in an airline domicile country (*ECONFREE*, expressed in logarithm), and being a global alliance member (*ALLIANCE*). These new determinants are tested alone and in combination with public ownership and/or political stability. Since we add a third input in the production function and given the limited size of our data set, we can provide evidence only for the Cobb-Douglas model, given that the translog function does not achieve convergence. We underline that only the results for the Colombi et al. (2014) model, with both persistent and transient efficiency, are presented in this Section.^16^ Table 5 reports the results for the different alternative specifications.

**Table 5:** Alternative specifications of the African airlines’ production frontier and determinants of transient and permanent inefficiency

	Dependent variable: $\log (RPK)$											
Independent variables	(1)	(2)	(3)	(4)	(5)	(6)	(7)	(8)	(9)	(10)	(11)	(12)
$\log (K)$	0.4063***	0.3113***	0.3769***	0.3132***	0.3177***	0.2415***	0.3209***	0.2589***	0.3794***	0.3136***	0.3166***	0.3606
	(0.0198)	(0.0177)	(0.0044)	(0.0082)	(0.0060)	(0.0076)	(0.0088)	(0.0133)	(0.0100)	(0.0390)	(0.0543)	(0.3372)
$\log (L)$	0.2090***	0.2242***	0.2376***	0.2673***	0.2448***	0.2730***	0.2453***	0.2734***	0.2413***	0.2742***	0.1579**	0.1446
	(0.0676)	(0.0354)	(0.0048)	(0.0114)	(0.0104)	(0.0095)	(0.0099)	(0.0260)	(0.0076)	(0.0594)	(0.0613)	(0.2704)
$\log (F)$	0.4293***	0.3978***	0.4878***	0.4302***	0.4916***	0.4284***	0.4903***	0.4273***	0.4875***	0.4350***	0.3843***	0.4028***
	(0.0226)	(0.0151)	(0.0056)	(0.0081)	(0.0053)	(0.0205)	(0.0165)	(0.0334)	(0.0032)	(0.0301)	(0.0722)	(0.1652)
*BETWEEN*	0.3324***	0.2466***	0.1834***	0.2116***	0.2639***	0.2594***	0.2756***	0.2571***	0.2016***	0.2278***	0.3982	0.2682
	(0.0159)	(0.0346)	(0.0029)	(0.0124)	(0.0063)	(0.0087)	(0.0067)	(0.0254)	(0.0030)	(0.0146)	(0.3133)	(0.1676)
$\overline{\log (K)}$		0.1487***		0.0167		0.1247***		0.0434*		0.0393*		−0.2639***
		(0.0103)		(0.0103)		(0.0062)		(0.0207)		(0.0195)		(0.0680)
$\overline{\log (L)}$		−0.1449***		−0.2465***		−0.2763***		−0.2582***		−0.2598***		0.1354
		(0.0136)		(0.0061)		(0.0142)		(0.0324)		(0.0448)		(0.1047)
$\overline{\log (F)}$		0.1746***		0.3097***		0.2739***		0.3328***		0.2879***		0.0374
		(0.0115)		(0.0084)		(0.0112)		(0.0269)		(0.0129)		(0.3918)
Constant	0.0117	0.1590***	0.0917***	0.0589***	0.0314*	0.0274***	0.0184*	0.0251	0.0711***	0.0436	−0.0427	0.1999
	(0.7660)	(0.0325)	(0.0030)	(0.0067)	(0.0149)	(0.0094)	(0.0090)	(0.0629)	(0.0064)	(0.0381)	(0.2254)	(0.1171)

	Factors affecting inefficiency											
	Time invariant inefficiency											
*PUB*	−6.6994***	−4.8872***					−2.1297***	−1.9476***	0.5095***	−0.0876		
	(0.0381)	(0.0231)					(0.0070)	(0.0098)	(0.0089)	(0.0550)		
*POLSTAB*	−5.9144***	−4.2305***									5.3221***	−1.900***
	(0.0457)	(0.0181)									(1.0851)	(0.3378)
*SUBSAHARA*	6.7358***	−5.9249***										
	(0.0272)	(0.0148)										
*ECONFREE*			−1.1583***	−0.0051					1.9024***	−4.6333***	−5.8328***	2.5513***
			(0.0049)	(0.0053)					(0.0045)	(0.0191)	(0.7757)	(0.1285)
*ALLIANCE*					−2.4735***	−2.3083***	9.6223***	10.7889***				
					(0.0071)	(0.0070)	(0.0076)	(0.0251)				
Constant	−19.4771***	6.7439***	−14.344***	−14.6797***	−10.0537***	−27.7775***	−17.4022***	−13.7873***	−26.2331***	−17.7405***	−2.2697***	−11.3872***
	(0.0524)	(0.0201)	(0.0049)	(0.0098)	(0.0106)	(0.0109)	(0.0058)	(0.0377)	(0.0039)	(0.0218)	(0.7014)	(0.4850)

	Time-varying inefficiency											
*PUB−*	0.0380	−0.0539*					−0.4240***	−0.4783***	−0.3387***	−0.3234***		
	(0.0316)	(0.0226)					(0.0282)	(0.0323)	(0.0062)	(0.0421)		
*POLSTAB*	0.4137**	0.2291***									2.3040***	2.5793***
	(0.1065)	(0.0165)									(0.4420)	(0.0580)
*SUBSAHARA*	3.9922***	2.4664***										
	(0.0415)	(0.0271)										
*ECONFREE*			−0.3947***	−0.6413***					−0.7559***	−0.5657***	−4.9622***	−0.7526***
			(0.0316)	(0.0097)					(0.0043)	(0.0129)	(0.1785)	(0.0963)
*ALLIANCE*					−1.8780***	−1.7883***	−1.9211***	−1.8217***				
					(0.0104)	(0.0182)	(0.0105)	(0.0214)				
Constant	−1.5610***	0.7336***	−0.0282***	−12.4228***	3.3370***	7.3804***	1.3756***	0.1850***	3.2040***	−11.3328***		42.3784***
	(0.0742)	(0.0189)	(0.0055)	(0.0107)	(0.0075)	(0.0113)	(0.0052)	(0.0501)	(0.0049)	(0.0491)		(0.2592)
log-likelihood	25.5711	31.2345	15.3628	19.5327	19.7494	25.3203	20.1792	25.7261	15.5988	19.8573	29.8175	20.5278

variables with overbar are Mundlak corrections

***0.1% significance level, **1% significance level, *5% significance level, ’10% significance level

Columns (1)–(2) show that input coefficients are as expected and that the coefficient of fuel is positive and significant. Interestingly, using *RPK* as the output variable, *BETWEEN* has a positive and significant coefficient (with the exception of the results shown in columns (11)–(12)). This implies that, when the output variable gives different weights to short-haul and long-haul flights, the association between H&S network structure and higher output volume is stronger. Regarding the determinants of inefficiency, these are, in columns (1)–(2), the same as our baseline model, i.e., *PUB*, *POLSTAB*, and *SUBSAHARA*. Looking at their estimated coefficients, and comparing them with the results for the Cobb-Douglas function shown in Column (5)–(6) of Table 3, we observe that *PUB* is confirmed as a factor decreasing both persistent and transient inefficiency, while political stability drives persistent inefficiency down and transient inefficiency up. *SUBSAHARA* is mainly confirmed as a driver of inefficiencies with the only exception of persistent inefficiency in column (2) of Table 5. In summary, the results in columns (1)–(2) of Table 5 provide a further confirmation (i) of the role of *PUB*, and (ii) of the fact that political stability has to be treated with care, since it may have different effects on transient efficiency, probably also due to the already discussed impossibility to discriminate between “good" and “bad" stability.

As discussed in Section 6, the results on *PUB* and *POLSTAB* may be driven by a general lack of competition, creating a favorable ground to protectionist practices (captured by *PUB*) and entrenched government (potentially incorporated in *POLSTAB*). It is therefore of interest to see the impact on the efficiency of a measure of a country’s economic freedom. This allows us to understand whether (i) the openness of an economy is a factor fostering airline efficiency also in the current African context, and (ii) if protectionism may have less predictive power as a determinant of inefficiencies when a measure of a country’s economic freedom is also incorporated in the analysis.^17^ Columns (3)–(4) of Table 5 present the estimated Cobb-Douglas production functions with *ECONFREE* as the only determinant of inefficiencies. There is strong enough evidence, and this is a first signal of the importance of this factor for the overall efficiency of African airlines, that economic freedom decreases both persistent and transient inefficiencies. The results shown in Columns (9)–(12) reinforce this insight. In columns (9)–(10) *ECONFREE* is confronted with *PUB* as determinant of inefficiencies. Interestingly, *PUB* is now either increasing persistent inefficiency, or it has no impact on it when we include the Mundlak correction in the Cobb-Douglas production function. On the contrary, there is strong evidence that *ECONFREE* decreases both persistent and transient inefficiencies. Hence, the degree of economic freedom seems more relevant in improving the African airlines’ efficiencies than the protectionism granted by the public umbrella.^18^

The same evidence is obtained if *ECONFREE* is confronted with a measure of entrenched government, a role (at least in part) played by political stability in our sample. The results in columns (11)–(12) of Table 5 confirm that the degree of economic freedom is decreasing both types of inefficiency, while *POLSTAB* is mainly a factor increasing inefficiencies, albeit the effect on persistent inefficiency is confirmed ambiguous. We, therefore, gather some evidence that, despite the peculiarity of the African context, improving competition may be the way forward for the development of an efficient air transportation system, at least for what concerns the airlines. This result has potentially noticeable implications in terms of future policy directions.

Columns (5)–(8) present the estimated Cobb-Douglas production functions (with and without Mundlak correction) when we include the participation in a global alliance as a determinant of inefficiencies. Columns (5)–(6) display that *ALLIANCE*, when it is the single factor affecting inefficiency, increases both persistent and transient efficiency. Columns (7)–(8) show instead that, when confronted with *PUB*, *ALLIANCE* increases persistent inefficiency while decreases transient inefficiency (with *PUB* exhibiting a positive impact on both efficiency types). Hence, on the one hand, *ALLIANCE* seems to have a weaker effect than economic freedom. While the latter is the main factor decreasing inefficiencies when it is confronted with *PUB*, the same relation is not observed with *ALLIANCE*. Indeed, the positive effect of *ALLIANCE* in providing enough incentives to improve short-run efficiency is robust to the inclusion of a factor capturing protectionism, while this does not happen for its effect on persistent efficiency.

**Fig. 5:**
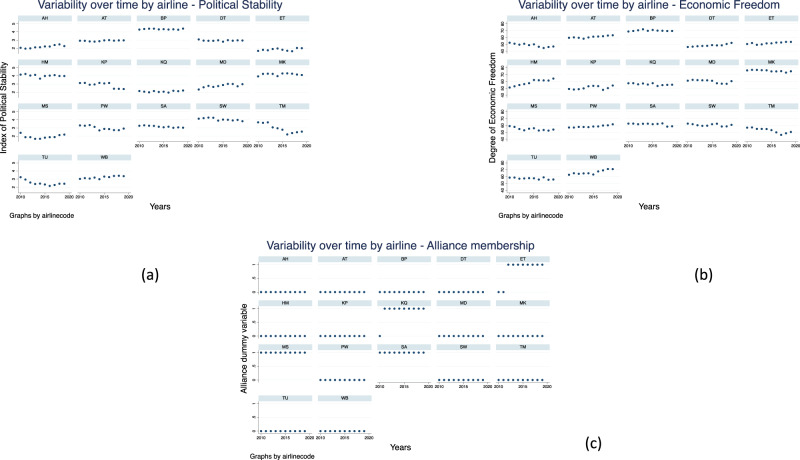
Variability of factors affecting efficiencies by airline. **a** Political Stability, **b** Economic Freedom, and **c** Alliance membership

Last, Fig. 5 in Appendix shows the variability of *ALLIANCE* (panel (b)), and *ECONFREE* (panel (c)) by airline. Their trends may explain the pattern of transient efficiency in Fig. 1. For instance, ET (*Ethiopian*) and KQ (*Kenya*) became a member of global alliances at the beginning of the observed period and they registered an improvement in transient efficiency later. Similarly, *ECONFREE* went up in Morocco, and *Royal Air Maroc* improved transient efficiency. The same is true for *Air Seychelles, HM*, and for *TAAG Angola Airways, DT*.

In summary, these additional insights suggest that the African air transportation system might benefit from being more open to the global market.

## Conclusions

In this paper, we estimate the technical efficiency of 17 African airlines during the period 2010-2019, i.e., before the crisis due to the COVID-19 outbreak. The production frontier is identified by implementing a state-of-the-art stochastic frontier model (Colombi et al. 2014, 2017) that has the error term decomposed into four random effect components: time-invariant persistent inefficiency, time-varying transient inefficiency, time-invariant airline’s unobserved heterogeneity, and random shocks. This model is compared with a nested model, defined by Greene (2005a, b) as a true random effect model, in order to appreciate the additional insights regarding the evaluation of technical efficiency obtained when unobserved heterogeneity is not confounded with time-invariant persistent inefficiency. To the best of our knowledge, this is the first paper that investigates African airline technical efficiency using the four random components stochastic frontier model. From the estimated production frontier each airline’s efficiency scores are computed, separated between persistent and transient efficiency, and analyzed according to some possible determinants of efficiency levels.

Based on this advanced stochastic frontier model we first estimate a baseline model with passengers as output and capital and labor as inputs; then we consider an alternative output variable, i.e., *RPK* that gives to passengers different weights according to the flown distance, and an additional input, i.e., fuel, that has to be included once that distance is taken into account. In the alternative specifications, we also compare different determinants of persistent and transient efficiencies. Using this empirical strategy we obtain some interesting results.

First, in the baseline model we find that as in Barros and Wanke (2015), public ownership is a factor improving both persistent and transient efficiency differently from what found in studies regarding non-African airlines (e.g., Yu et al. 2019). In our view, this highlights the importance in the African context of the protection granted by the public umbrella. However, in an alternative specification, where also economic freedom is taken into account and confronted with public ownership, it turns out that economic freedom is a more robust and important driver of airline efficiency than protectionism, whose positive effect on persistent efficiency disappears. Hence, we gather some evidence that, despite the peculiarity of the African context, improving competition may be the way forward for the development of an efficient air transportation system, at least for what concerns the airlines. This result has potentially noticeable implications in terms of future policy directions. Second, in our baseline model we find that a country’s political stability may lead to higher transient efficiency but to lower persistent efficiency. As discussed in the paper, political stability in Africa is not always a condition leading to reforms that may shape the economy toward market efficiency: it may instead be linked to the existence of entrenched governments. Indeed, in the alternative specifications of our model, where we confront political stability with economic freedom, we find that the latter has a more clear and positive effect on efficiency than the former, suggesting, again, the importance of reforms implementing an effective competition in the African air transportation sector.

Third, we find that Mediterranean countries have more efficient airlines than those with headquarters located in sub-Sahara, and this finding is reasonably due to both open sky agreements with Europe (e.g., Morocco), and more intense competition coming from major European airlines. Fourth, concerning the importance of inputs in the estimated production, we find that the output elasticity of capital (i.e., total seats available in the airline fleet) is higher than that of labor (+0.51% versus +0.07%). This, combined with evidence that the marginal product of capital is always positive for all observations in our data set, confirms the importance of the optimal use of durable capital inputs for African airlines. Last, we find evidence of decreasing returns to scale, of an average persistent efficiency equal to 78%, and of a mean of transient efficiency of 80%. This combined evidence implies that efficiency in African airlines is low and that it is important to remove the conditions that make air carriers operate inefficiently.

In summary, this paper provides an empirical base, obtained with advanced econometric methods, for supporting the completion of the liberalization of air transportation in Africa. The first step in this process may be the full implementation of SAATM (and YD), as well as more open sky agreements, especially with Europe.

There are some possible extensions to the analysis performed in this paper: increase the number of African airlines, increase both the output and the input variables, and compare African and non-African airlines. They are left for future research.

## Appendix

Figure 5

## Footnotes

1 They find that Asian airlines are more efficient that European and North American ones.

2 Even if not focused on aviation, another interesting contribution estimating persistent and transient efficiency in Africa is Adom et al. (2018), which studies energy efficiency for African countries.

3 As shown by Colombi et al. (2014), Proposition 1, under assumptions (A1)–(A5), the vectors of outputs ${{{{\boldsymbol{y}}}}}_{i}={({y}_{i1},{y}_{i2}\ldots ,{y}_{iT})}^{{\prime} },i=1,2,\ldots ,n,$ are independent and have a Closed Skew Normal (CSN) density. The maximization of the log-likelihood of the model (1) and ML estimators are discussed in Colombi et al. (2014), Proposition 2, which also showed (Proposition 3) how to compute the efficiency scores $E[\exp (-{u}_{i})| {{{{\boldsymbol{y}}}}}_{i}\left.\right)]$ and $E[\exp (-{u}_{it})| {{{{\boldsymbol{y}}}}}_{i}\left.\right)]$ for each airline *i* and period *t*.

4 The Cobb-Douglas production function is popular and easier to estimate (fewer parameters involved). However, it has low flexibility since the input elasticity of substitution (i.e., the ratio between two input and their marginal products) is fixed. On the contrary, the translog production function is flexible and does not have a constant elasticity of substitution, but it might have problems of convergence in the ML estimation because it has a larger number of independent variables.

5 While political stability in developed countries has a positive impact on economic growth, because it allows implementing unpopular reforms aimed at market efficiency and elimination of rents, in African countries it may be the outcome of dictatorship or some form of monopoly in the political system, giving rise to favoritism and accumulation of rents.

6 The Association members represent over 85% of total international traffic carried by African airlines (AFRAA 2020).

7 In case of missing data gaps are filled by interpolating the values of previous and following years and integrating data from the Official Airline Guide (OAG).

8 Each report refers to the previous year’s data.

9 *B**E**T**W**E**E**N*_*i**t*_ is computed using the information on each carrier’s airport pairs extracted from the OAG schedule analyzer. We consider routes having, on average, at least one flight per week in a year.

10 Almost all flights of *Air Seychelles* originate or land in Seychelles International Airport.

11 The year of the first flight is taken from different sources, mainly information available in the aircraft manufacturer website and on data available in internet.

12 See the website https://blog.openairlines.com/how-much-fuel-per-passenger-an-aircraft-is-consuming, access 8*th* February 2023.

13 We are grateful to an anonymous referee for suggesting this point.

14 In the two inputs case the direct elasticity of substitution coincides with the Allen elasticity of substitution.

15 We are grateful to an anonymous referee for this suggestion.

16 We had also to reduce the number of determinants of efficiencies compared to the baseline model presented in Table 3. Due to these limitations, we do not use these estimations as our main results. However, we believe that some of the new insights obtained can be of interest and may complement the findings discussed in Section 6.

17 We are grateful to an anonymous referee for this suggestion.

18 These results also suggest that despite protectionism may result in short-term advantages (it decreases transient inefficiency), they may disappear in the long run.
